# Eye of the Tiger Sign in Pantothenate Kinase-Associated Neurodegeneration

**DOI:** 10.1155/2021/6633217

**Published:** 2021-05-07

**Authors:** S. Choayb, H. Adil, Daoud Ali Mohamed, N. Allali, L. Chat, S. El Haddad

**Affiliations:** Children's Hospital, Radiology Department, Mohamed V University, Rabat, Morocco

## Abstract

Pantothenate kinase-associated neurodegeneration (PKAN) is a rare disorder associated with brain iron accumulation caused by a recessive mutation in pantothenate kinase 2 gene (PANK2). We present a case of an 11 year-old boy presenting extrapyramidal signs and developmental regression. T2-weighted images showed the classic eye of the tiger sign seen in pantothenate kinase-associated neurodegeneration.

## 1. Introduction

Neurodegeneration with brain iron accumulation (NBIA) is a group of diseases characterized by an abnormal accumulation of iron in the basal ganglia leading to various progressive disorders of movement and development. There are ten main types, including pantothenate kinase-associated neurodegeneration (PKAN), which is the most common form [1]. The classic appearance helping in the diagnosis is the eye of the tiger on T2-weighted-images (hypointensity of the globus pallidus with a central hyperintensity).

## 2. Case Report

An 11 year-old boy from a consanguineous marriage with no significant past medical history presents at the age of 9 psychomotor delay, upper limb tremor, and frequent falls. There was no family history of neurological diseases. Clinical examination revealed dysarthria, spastic paraparesis with rigidity, and dystonic movements of the upper member. Laboratory investigations and EEG were normal.

**Figure 1:**
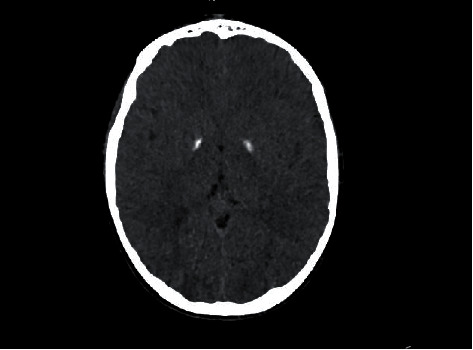
Axial CT scan showing bilateral calcifications of the globus pallidus.

An initial head CT showed bilateral calcifications of the globus pallidus (Figure 1).

**Figure 2:**
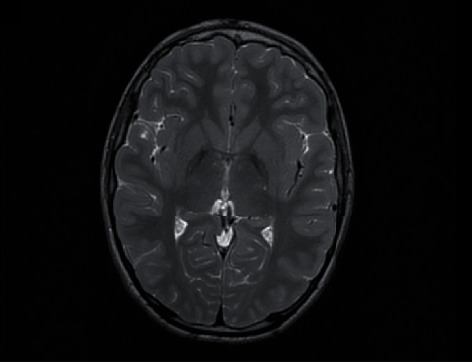
Axial T2WI: symmetric areas of low signal T2 of the globus pallidus with relatively central hyperintensity giving the appearance of eyes of a tiger.

**Figure 3:**
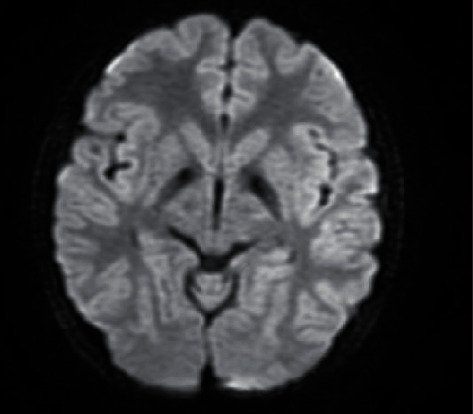
Axial T2∗WI: showing hypointense signal in the corresponding areas.

A brain MRI was performed, revealing diffuse bilateral and symmetric hypointensity of both the globus pallidus with relatively central hyperintensity in T2-weighted images (Figure 2). These areas showed susceptibility artifacts (low signal) in T2∗ sequences (Figure 3). No other signal alterations were observed in other regions of the basal ganglia. These findings were consistent with the eye of the tiger sign that is characteristic of pantothenate kinase-associated neurodegeneration but not pathognomonic.

## 3. Discussion

PKAN formerly known as Hallervorden-Spatz disease is a neurodegenerative brain disorder characterized by brain iron accumulation that is caused by a recessive mutation in the pantothenate kinase 2 gene (PANK2).

The PANK2 is necessary for the production of coenzyme A in mitochondria. Abnormal PANK2 function will lead to an accumulation of N-pantothenyl cysteine and free cysteine. Cysteine is a potent iron chelator and can lead to secondary iron accumulation that will exacerbate neuronal injury by inducing oxidative stress [2].

The amount of residual enzyme activity influences the disease severity; patients with 2 null mutations (resulting in absent PANK2 enzyme) consistently have the earlier disease onset and later disease onset in individuals with mutations that allow residual enzyme activity. Globally, the 1561G>A missense mutation is the most common cause of PKAN, and homozygotes have classic PKAN [2].

In the classical form, the first manifestations appear in infancy, between 3 and 4 years old, and progress rapidly [2, 3]. The clinical presentation is characterized by progressive extrapyramidal dysfunction; dystonia is a prominent feature of this disorder and affects the cranial region and the limbs. In cases of oromandibular dystonia, it is often followed by speech difficulties and dysarthria. Other features include corticospinal tract involvement (hypertonicity, hyperreflexia, spasticity, and upgoing plantar responses), optic atrophy, retinopathy pigmentosa that may lead to cataracts, and acanthocytosis.

Atypical PKAN is characterized by later onset (age > 10 years), motor involvement tends to be less severe, but cognitive decline and psychiatric alterations are predominant traits. Speech difficulties (such as palilalia or dysarthria), psychiatric problems, behavioral difficulties, or frontotemporal-like dementia early in their disease are common presenting features.

Extrapyramidal dysfunction, corticospinal tract signs, and retinitis pigmentosa are also described, but they are less prevalent than in the classic form [2, 4, 5].

On MRI, eye of the tiger sign is the typical manifestation. T2-weighted sequences show hypointensity of the globus pallidus with a central hyperintensity, reflecting an excessive accumulation of iron with gliosis.

Hypointensity of the substantia nigra may be present in some cases. Eye of the tiger sign is not pathognomonic of PKAN although the majority of individuals carry PANK2 mutations [3, 6].

The caliber and intensity of the eye are predominant in early symptomatic patients. As the disease progresses, pallidal hypointensity increases [2].

This sign has also been reported in other diseases (neurodegeneration associated with mitochondrial membrane proteins, multiple system atrophy, carbon monoxide poisoning, aceruloplasminemia, and neuroferritinopathy) and should be interpreted correctly in the clinical context. PANK2 genetic testing is the gold standard for confirming the diagnosis [3, 6].

In our case, molecular analysis of the PANK2 gene revealed a missense homozygous mutation in exon 2.

CT is not used in routine for patients suspected of PKAN. Thus, the incidence of basal ganglion calcifications may be underestimated [7]. Eye of the tiger sign with bilateral basal ganglia calcifications has also been reported previously in one similar patient, by Fasano et al. [8].

In our country, PKAN was first described in a 10-year-old girl born from first-degree consanguineous parents with PANK2 homozygous deletion. She had a typical PKAN phenotype presentation with extrapyramidal signs and developmental regression at the age of 7. Brain MRI performed at the age of 9 revealed the classic eye of the tiger sign. Molecular analysis identified a rare homozygous deletion in PANK2 exon 3 [9].

Our patient is the first case in Morocco demonstrating both eye of the tiger sign on MRI and bilateral basal ganglia calcifications on CT.

The natural history of classical PKAN is a nonuniform progression with intervals of stability interspersed with periods of neurological deterioration leading to early adulthood death.

Atypical PKAN is less aggressive than the classic disease, and most individuals remain ambulant until adulthood. There is presently no cure. Current management strategies focus on medical and surgical symptom palliation to improve the quality of life. [3]
